# Transarterial embolization for falx dural arteriovenous fistula through the artery of Davidoff and Schechter: A case report

**DOI:** 10.1016/j.radcr.2021.12.013

**Published:** 2021-12-28

**Authors:** Tomohiro Sugiyama, Yosuke Tajima, Yoichi Yoshida, Toshiki Ishikura, Yasuo Iwadate

**Affiliations:** Department of Neurological Surgery, Graduate School of Medicine, Chiba University, Inohana 1-8-1, Chuo-ku, Chiba, 260-8670, Japan

**Keywords:** TAE, transarterial embolization, TVE, transvenous embolization, DAVF, dural arteriovenous fistulae, ADS, the artery of Dovidoff and Schechter, Dural arteriovenous fistulae, Transarterial embolization, Artery of Davidoff and Schechter

## Abstract

Endovascular transarterial embolization is the standard treatment for the nonsinus-type of dural arteriovenous fistulae. Here, we report a rare case of successful transarterial embolization from the artery of Davidoff and Schechter for falx dural arteriovenous fistulae. A 74-year-old-man was incidentally diagnosed with falx dural arteriovenous fistulae during head magnetic resonance imaging. Results revealed dilatation of the cortical veins in the right occipital lobe. Angiographically, falx dural arteriovenous fistula was observed to be fed by the right middle meningeal artery, right occipital artery, right posterior meningeal artery, and the artery of Davidoff and Schechter (Borden type III). However, due to the tortuosity, the first transarterial embolization surgery through the middle meningeal artery, occipital artery, and posterior meningeal artery was unsuccessful. Therefore, the second transarterial embolization was performed through the artery of Davidoff and Schechter. Arteriovenous fistulae disappeared after administering Onyx injections through the artery of Davidoff and Schechter. Based on our findings, the artery of Davidoff and Schechter can be an approach route to treat dural arteriovenous fistulae. Moreover, the most important point of transarterial embolization procedures through the artery of Davidoff and Schechter is to navigate the microcatheter along the falx.

## Introduction

Intracranial dural artereriovenous fistulae (DAVF) account for approximately 10%–15% of intracranial arteriovenous malformations [1]. Although the arterial supply is frequently from adjacent meningeal branches, its venous drainage can be highly variable [2]. Therefore, surgical or endovascular therapy strategies are recommended for the cases of aggressive type of DAVF that shows cortical venous drainage due to the high risk of intracranial hemorrhage [3]. In the sinus type of DAVF, endovascular transvenous embolization is the standard treatment. However, endovascular transarterial embolization (TAE) is the standard treatment for the nonsinus-type of DVAF [3]. Here, we report a rare case of successful TAE for falx DAVF from the artery of Davidoff and Schechter (ADS), which is a meningeal branch of the posterior cerebral artery (PCA).

## Case report

**Fig. 1:**
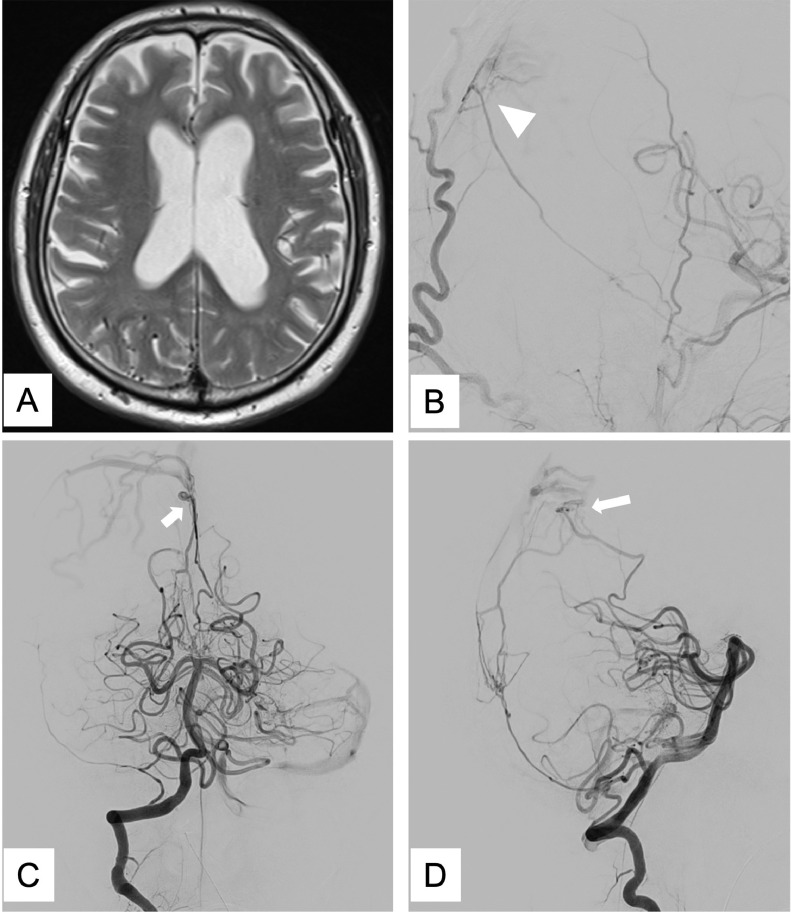
T2-weighted imaging showing the dilatation of cortical veins at the right occipital lobe (A). The right external carotid angiography image of the lateral view showing the arteriovenous fistula on the falx (arrowhead) (B). Right vertebral angiography showing a similar fistula (arrow) (C, D). C: anteroposterior view, D: lateral view

A 74-year-old-man was incidentally diagnosed with falx DAVF during head magnetic resonance imaging (MRI). The patient had no abnormal neurological symptoms and no history of trauma. However, MRI revealed dilatation of the cortical veins in the right occipital lobe (Fig. 1A). Therefore, cerebral angiography was conducted because an arteriovenous shunt was suspected. The right external cerebral angiogram demonstrated an arteriovenous fistula on the falx that was fed by the petrosquamous branch of the middle meningeal artery (MMA) and the trans-osseous branch of the occipital artery (OA) (Fig. 1B). Additionally, the right vertebral angiogram showed a similar fistula fed by the posterior meningeal artery (PMA) and ADS arising from the left PCA (Figs. 1C andD). However, the internal carotid angiogram revealed no feeding arteries. Moreover, the draining routes of arteriovenous fistula were cortical veins in the parietal and occipital lobes, with accompanying venous congestion. Therefore, the patient was diagnosed with falx DAVF (Borden type III) that had a high risk of intracranial hemorrhage. Hence, we tried occluding the fistula through TAE because of its nonsinus nature and considered approaching it via the MMA as the first route. If infeasible, we planned to approach it via the PMA as the second route.

**Fig. 2:**
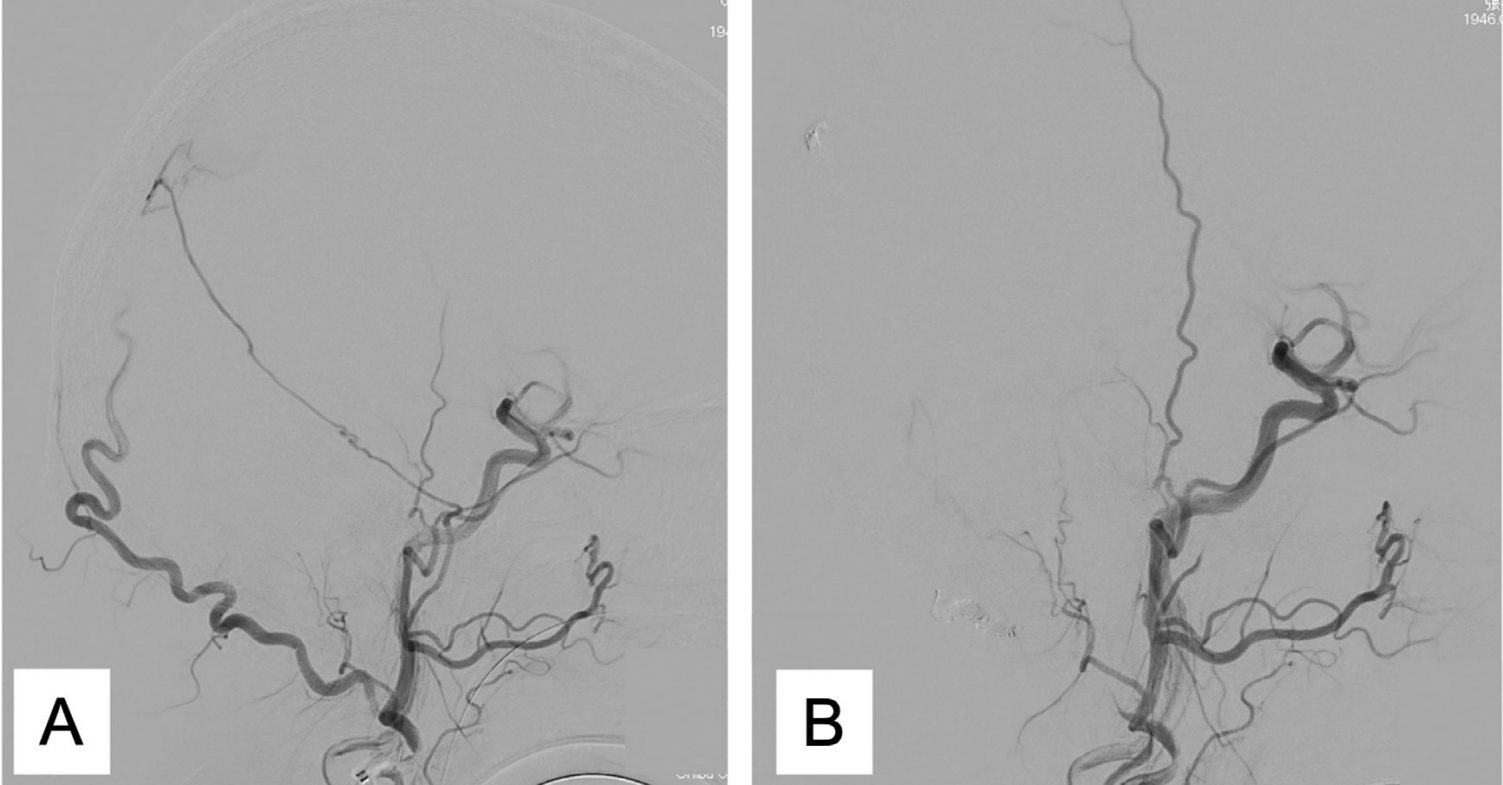
Initial angiogram from the right external carotid artery showing the dural arteriovenous fistula fed by the petrosquamous branch of the middle meningeal artery [MMA] and the trans-osseous branch of the occipital artery [OA]. An angiogram from the right external carotid artery after transarterial embolization showing the disappearance of the dural arteriovenous fistula (A).

**Fig. 3:**
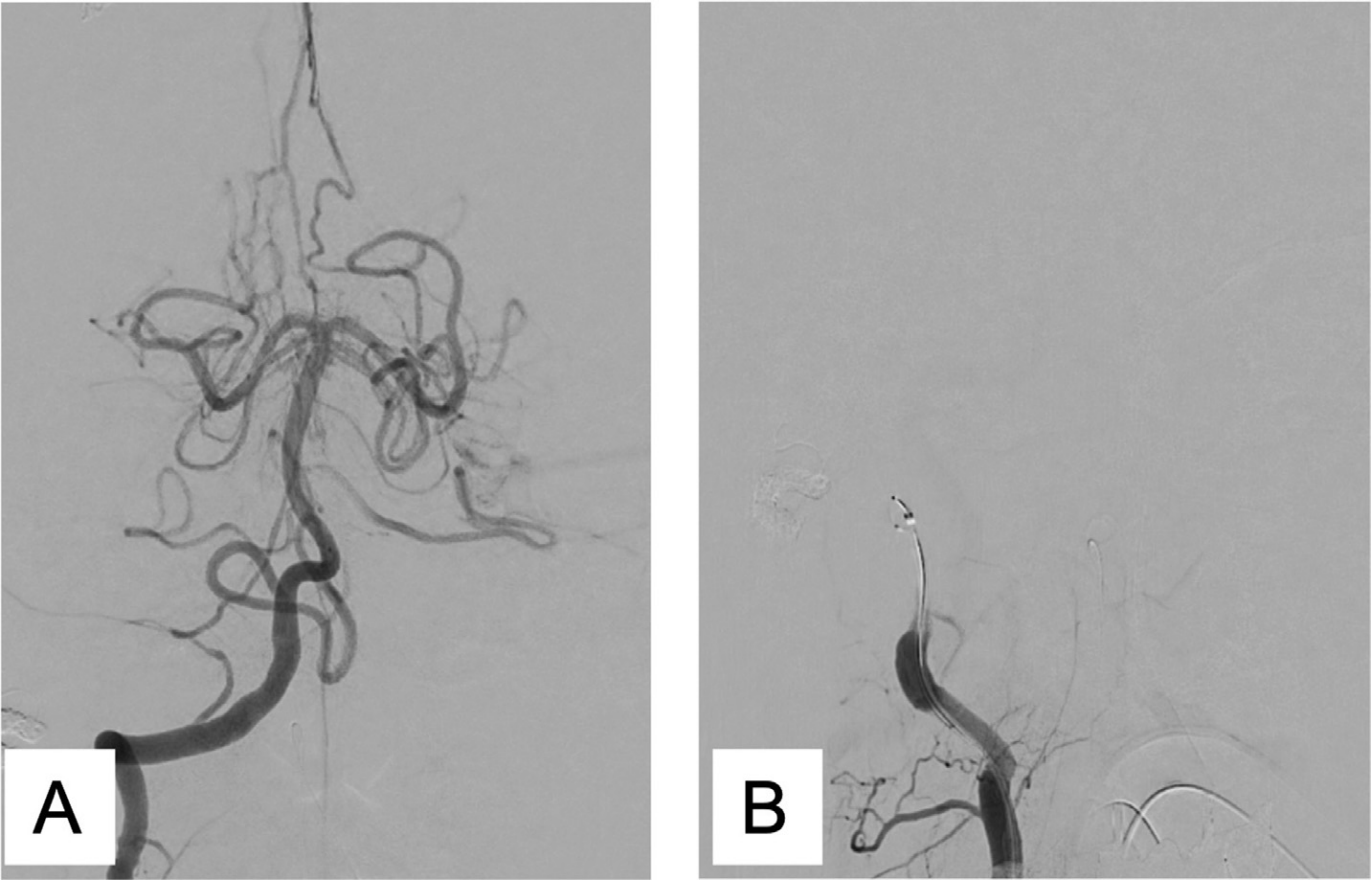
Right vertebral angiography shows residual dural arteriovenous fistula fed by the right posterior meningeal artery [PMA] and the artery of Davidoff and Schechter (A). After trying to introduce a microcatheter at the right PMA, the vertebral artery got dissected (B)

**Fig. 4:**
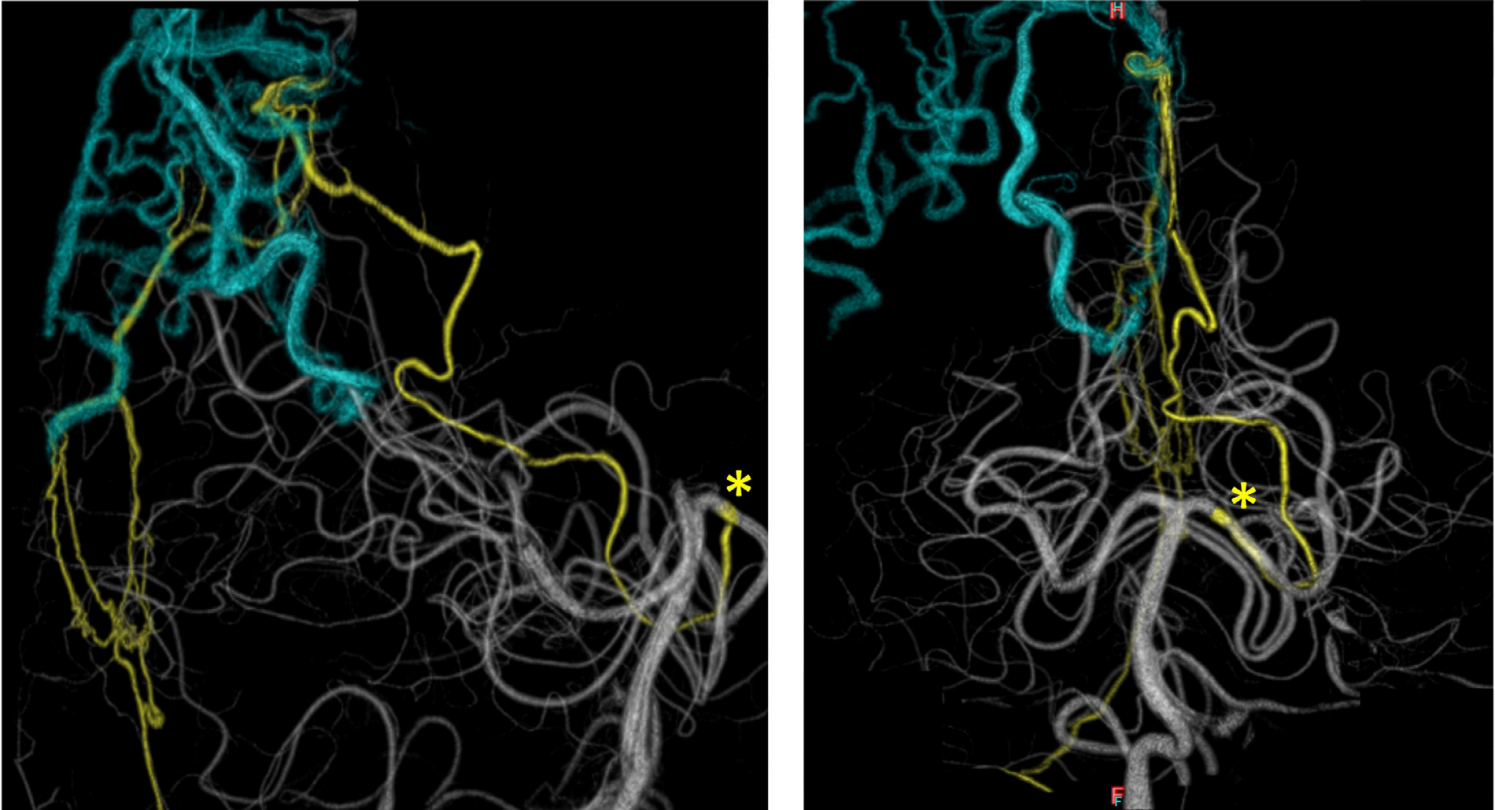
A 3-dimensional rotational angiography of the left vertebral artery showing details of the course of the artery of Davidoff and Schechter [ADS]. (A) lateral view, (B) anteroposterior view. The asterisk indicates the origin of ADS

TAE was conducted under general anesthesia. A 4F FUBUKI dilator kit of 90 cm (ASAHI INTECC, Aichi, Japan) was introduced in the right external carotid artery. The headway (Microvention, Tustin, CA, USA) was then replaced at the second portion of the right OA with CHIKAI (ASAHI INTECC). Since the right OA was tortuous, we were unable to introduce headway to the shunt point. Hence, we placed some platinum coils for feeder occlusion (Figs. 2A andB). Subsequently, a Marathon catheter (Medtronic, Dublin, Ireland) was replaced in the petrosquamous branch of the right MMA. A 25% N-butyl-2-cyanoacrylate (NBCA)–lipiodol mixture was also injected into the fistula. However, we were unable to achieve penetration into the fistulous connections, which resulted in incomplete obliteration (Figs. 2A and B). Hence, we tried introducing a DeFrictor Nano Catheter (Medico's Hirata, Osaka, Japan) to PMA. However, during the cannulation, a dissection in the right vertebral artery (VA) occurred (Fig. 3A). Therefore, we conducted the parent artery occlusion of the right VA using platinum coils (Fig. 3B). Post treatment, 3-dimensional rotational angiography (3DRA) of the left VA demonstrated the detailed course of ADS (Figs. 4A and B). Due to the weak tortuousness of ADS, we selected ADS as the next pathway.

**Fig. 5:**
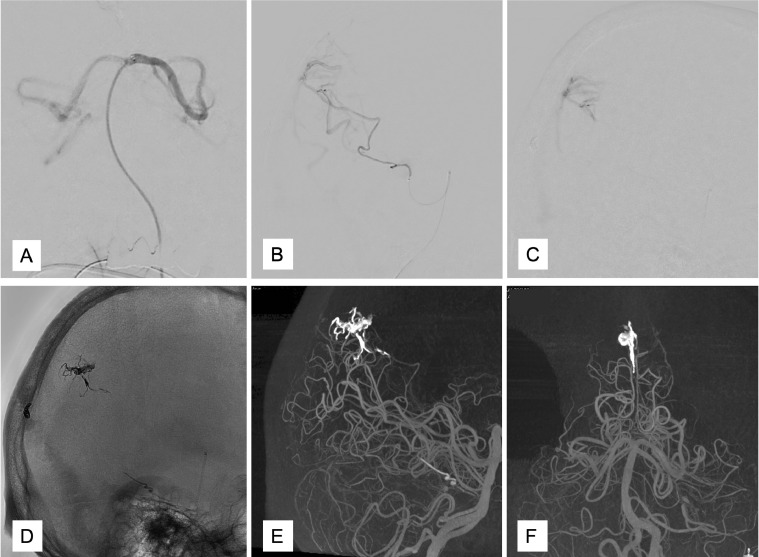
GuidePost was placed in P1 of the left posterior cerebral artery (A). The Marathon catheter was navigated into the artery of Davidoff and Schechter (B). The Marathon reached the shunt point (C). Spot lateral radiograph of the skull showing the Onyx cast, following transarterial embolization (TAE) (D). The minimum intensity projection image observed after left vertebral artery injections and TAE showing no residual early venous filling, with an Onyx cast filling the cortical vein (E, F). (E) lateral view, (F) anterolateral view

**Fig. 6:**
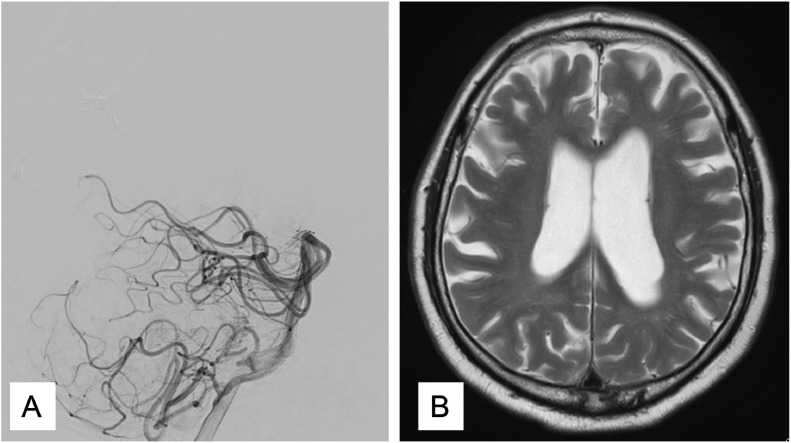
T2-weighted imaging showing no abnormal vessels (A). The lateral view of the left vertebral angiography showing no recurrence of the falx dural arteriovenous fistula (B)

Three months later, we tried TAE via ADS. A 4F FUBUKI dilator kit was placed in the left VA. Then, GuidePost (Tokai Medical, Aichi, Japan) was placed in P1 of the left PCA (Fig. 5A). Subsequently, we navigated a Marathon catheter into ADS (Fig. 5B) and reached the shunt point (Fig. 5C). Afterwards, we injected Onyx (Covidien, Irvine, CA, USA), causing a penetration to the venous portion (Fig. 5D), following vanishing of the shunt (Figs. 5E and F). Post treatment MRI revealed the disappearance of dilatation of the cortical veins in the right occipital lobe (Fig. 6A). Follow-up digital subtraction angiography also showed complete obliteration and no recurrence (Fig. 6B).

## Discussion

The most important point accounting for a successful TAE is to navigate the microcatheter to the shunt point as close as possible. Here, we used the petrosquamous branch of MMA as an approach route first. Then, we used the PMA. However, as both the arteries were tortuous, our attempts were unsuccessful. Therefore, we chose ADS as the third pathway for the shunt point.

ADS arises from the peduncular or ambient segment of the PCA [4]. This artery supplies the medial aspect of the tentorium and the posterior portion of the falx. It also extends posterolaterally across the ambient cistern between the superior cerebellar artery superiorly and trochlear nerve inferiorly [4]. ADS is so small that it is rarely seen on angiography. However, it is seen in cases where it had become enlarged, such as when it supplies a posterior fossa of DAVF, vascular mass in the posterior part of the septum pellucidum, meningioma of the tentorial incisural region, or a cerebellar hemangioblastoma [5, 6].

Here, the angle of ADS origin was not too acute. Therefore, we were unable to navigate the Marathon to ADS with ease. Moreover, the tortuousness of ADS itself was not strong. Owing to the above factors, ADS was a good approach route. Therefore, direct ADS communication between the dural arterial network and the intradural posterior circulation poses a potential risk of iatrogenic stroke related to the reflux of liquid embolic agents toward the pial vessel [7]. Nevertheless, during the embolization of ADS, care should still be taken to assess the reflux back toward the PCA and basilar artery. Hence, we propose that the most important point of the successful TAE conducted via ADS was to navigate the microcatheter through the falx.

In conclusion, we present a rare case of falx DAVF treated through the TAE strategy via ADS. ADS can be an approach route to cure DAVF. However, the most important point accounting for TAE via ADS was to navigate the microcatheter through the falx. The 3DRA is also useful to recognize details of the course through ADS.

## Patient consent

Informed consent was obtained from the patient for the publication of this case report and accompanying images.
